# Protection against myocardial ischemia reperfusion injury by liquiritin: involvement of autophagy restoration targeting PIK3CA

**DOI:** 10.1590/1414-431X2025e14964

**Published:** 2026-01-09

**Authors:** Huizi Mao, Zhuqing Li, Chengzhi Lu

**Affiliations:** 1 First Central Hospital of Tianjin Medical University, Tianjin, China; 2 Department of Cardiology, The First Affiliated Hospital of Jinzhou Medical University, Jinzhou, Liaoning, China; 3 Department of Cardiology, Tianjin First Central Hospital, Tianjin, China

**Keywords:** Liquiritin, Myocardial ischemia reperfusion injury, PIK3CA, Autophagy, Apoptosis

## Abstract

The current therapy for myocardial infarction focuses on reestablishing blood flow in the coronary arteries to reduce the ischemic area, but the subsequent damage caused by reperfusion cannot be ignored. Liquiritin, a primary flavonoid compound found in the medicinal plant licorice, exhibits distinct pharmacological properties including neuroprotection, anti-inflammatory, antioxidant, and anti-apoptotic effects. However, further research on its role and mechanism in myocardial ischemia-reperfusion (I/R) injury is needed. The aim of this work was to elucidate the protection of liquiritin against myocardial I/R insult and whether liquiritin-mediated autophagy restoration was associated with phosphatidylinositol-4,5-bisphosphate 3-kinase catalytic subunit alpha (PIK3CA) *in vivo* and *in vitro*. Liquiritin administration by oral gavage inhibited pathological injury of myocardial I/R injured rats, evidenced by improved cardiac function and reduced infarct size. Moreover, liquiritin restored excessive autophagy by promoting the phosphorylation of protein kinase B (AKT) and mammalian target of rapamycin (mTOR), which was accompanied by PIK3CA upregulation. Mechanistically, silencing PIK3CA in rat *H9c2* cardiomyoblasts diminished the beneficial effects against oxygen-glucose deprivation/reoxygenation (OGD/R) injury reflected by exacerbated apoptosis and dysregulated autophagy mediated by the classical PI3K/Akt/mTOR pathway. Liquiritin inhibited excessive autophagic flux via decreasing autophagosome-lysosome fusion, which was similar to the effect of the autophagy inhibitor chloroquine. Moreover, this phenomenon was enhanced when liquiritin and chloroquine were used in combination. Collectively, our work revealed that the protective effect of liquiritin against myocardial I/R injury may be attributed to its autophagy restoration mediated by PIK3CA.

## Introduction

Myocardial infarction, one of the most severe cardiovascular diseases, is caused by a blockage or reduced blood flow to a specific area of the myocardium (1). Among cardiovascular diseases, coronary heart disease (CHD)-induced myocardial infarction is a leading cause of mortality (2). At present, the treatment options for myocardial infarction mainly include reperfusion strategy and pharmacological therapy (3). Reperfusion strategy aims to restore blood flow in the coronary arteries and can be achieved through percutaneous coronary intervention and surgical coronary artery bypass grafting (4,5). Although these measures are beneficial to blood flow recovery, which limits myocardial ischemia, reperfusion itself paradoxically exacerbates myocardial damage, known as ischemia-reperfusion (I/R) injury (6,7). Understanding the mechanism during myocardial I/R injury may offer a valuable insight into the development of new therapeutic strategies.

Myocardial I/R injury typically leads to myocardial necrosis and the mechanisms involved encompass various processes, including autophagy (8), apoptosis activation (9), mitochondrial dysfunction (10), and endoplasmic reticulum stress (11). Previous studies primarily focused on the general pathophysiological responses in myocardial I/R injury; however, recent attention has turned to autophagy, a cellular lysosomal degradation process unique to eukaryotes (12,13). Autophagy is crucial for maintaining cellular homeostasis and is up-regulated under stress (14,15). Impaired or excessive autophagy contributes to pathological processes like myocardial I/R injury, where upregulated autophagy is linked to cardiomyocyte death (16,17). Inhibition of autophagy is shown to suppress myocardial I/R injury-related cell apoptosis and provide protection for cardiomyocytes against the insult of myocardial I/R (18). Hence, gaining a better understanding of the roles of autophagy in myocardial I/R injury may offer valuable insights for the development of therapeutic strategies.

Phosphatidylinositol-4,5-bisphosphate 3-kinase catalytic subunit alpha (PIK3CA, also known as PI3Kα) is the catalytic subunit p110a of phosphoinositide 3-kinase (PI3K), which functions as a lipid kinase acting as a key mediator of growth factor signaling through membrane receptor tyrosine kinases (RTKs) (19,20). The protective roles of PIK3CA in models of heart disease have been confirmed by previous research (21,22). The function of PIK3CA in the heart has been extensively studied using a kinase null dominant negative model (PI3KαDN). Except for a decrease in cardiomyocyte and heart size in PI3KαDN mice, there is an impaired response to pressure overload and a lack of physiological hypertrophy during swim training (21). Although the findings indicate the cardioprotective effect of PIK3CA, its role and mechanism in the myocardial I/R injury process requires further study.

Given the critical role of PIK3CA in cardiac survival signaling and the modulation of autophagy, compounds capable of targeting this axis warrant investigation. Liquiritin is one of the main components of licorice, a plant classified under medicine-food homology (23). The pharmacological properties include neuroprotection, anti-apoptosis, anti-oxidation, and anti-inflammation (24). The previous published works have demonstrated that liquiritin exerts protective effects against glutamate-induced cell damage in the differentiated rat pheochromocytoma cells (25). Furthermore, previous studies have confirmed that incubation in liquiritin protects human brain microvascular endothelial cells from apoptosis induced by oxygen-glucose deprivation/reoxygenation (OGD/R), an established *in vitro* model mimicking ischemia-reperfusion *in vivo* (26). In addition, the protective effect of liquiritin against cerebral I/R injury has been verified in mouse models (27). It is worth noting that activating the PI3K/protein kinase B (AKT) signaling pathway is a pivotal mechanism underlying liquiritin's diverse protective effects on cell differentiation, proliferation, survival, and apoptosis (25). A recently published study has demonstrated that liquiritin ameliorated OGD/R-induced cardiomyocyte injury based on network pharmacology (28), while its role in myocardial I/R injury remains unexplored, particularly in the context of autophagy modulation via the PI3K/AKT/mammalian target of rapamycin (mTOR) axis.

The aim of this study was to describe the cardioprotective effect of liquiritin in rats subjected to myocardial I/R injury and assess the related mechanism in the OGD/R injured *H9c2* cell line, which may provide a theoretical basis for myocardial I/R injury.

## Material and Methods

### Ethics approval

All animal experiments complied with the ARRIVE guidelines and were conducted according to the U.K. Animals (Scientific Procedures) Act, 1986, EU Directive 2010/63/EU, and the NIH Guide for the Care and Use of Laboratory Animals (NIH Publication No. 8023, revised 1978). All animal care and laboratory procedures strictly observed the Guide for Care and Use of Laboratory Animals and were approved by the Animal Ethics Committee of Jinzhou Medical University (No. 202305012).

### Animal treatment schedule

Male Sprague-Dawley rats weighing 220-280 g were supplied by the Liaoning Changsheng Biotechnology Co., Ltd. (License: SCXK (Liao) 2019-0007). All rats were acclimatized to the laboratory housing conditions (22±1°C, 45-55% humidity, and 12-h light/dark cycle) for a week prior to experiments. The rats were randomly divided into four groups: sham-operation (Sham), myocardial I/R injury (I/R), I/R + 20 mg/kg liquiritin (I/R + liquiritin-L), and I/R + 40 mg/kg liquiritin (I/R + liquiritin-H). The rats in I/R + liquiritin-L and I/R + liquiritin-H groups were orally administered with liquiritin at doses of 20/40 mg/kg (dissolved by 0.5% carboxymethylcellulose sodium solution) for three consecutive days and the remaining rats received an equivalent volume of vehicle (0.5% carboxymethylcellulose sodium solution). Myocardial I/R injury surgery was carried out 1 h after the last liquiritin administration. The rats were anesthetized with isoflurane (3% isoflurane for induction and 2% isoflurane for maintenance), the left anterior descending coronary artery was ligated with 6-0 thread. Thirty minutes after the ligation, the suture was gently loosened and reperfusion lasted for 2 h. The sham rats underwent only thoracotomy without the ligation. Subcutaneous injection of 2 mg/kg meloxicam was used to control postoperative pain. All animals were euthanized 2 h after reperfusion using a 50% volume per min displacement rate of CO_2_. The heart and blood were collected for the following analyses.

### Electrocardiogram (ECG) and echocardiography

ECG and heart rate (before and after the ligation at the left anterior descending coronary artery) were recorded using the MD3000 biological signal acquisition system (Beijing Zhishu Duobao Biological Technology Co., Ltd., China).

Echocardiography detection was carried out with Vevo 3100 system (FujiFilm, Canada) by analyzing the M-mode images. Under anesthesia with isoflurane inhalation, the parameters of left ventricular ejection fraction (LVEF), left ventricular internal dimension at end-diastole (LVIDd), left ventricular internal dimension at end-systole (LVIDs), and fractional shortening (FS) were recorded.

### Triphenyltetrazolium chloride (TTC) staining

The hearts were collected 2 h after reperfusion, frozen in liquid nitrogen for 20 s, and cut into slices with a thickness of 1-2 mm. The heart slices were stained by TTC solution (Sigma, USA) to assess myocardial infarct size after I/R. Briefly, the samples were immersed in TTC solution at 37°C for 30 min and fixed in 4% paraformaldehyde solution (Beyotime Biotechnology, China) for 24 h in turn. The infarcted area remained unstained, while the non-infarcted tissue was stained red. Image-Pro Plus 6.0 (USA) was employed in image analysis. The percentage of myocardial infarct was calculated as infarct area/whole heart area × 100%.

### Biochemical indicators

The serum was obtained by centrifugation at 1006 *g* for 10 min at 4°C and used to evaluate the biochemical alterations, including creatine kinase isoenzyme (CK-MB), cardiac troponin I (cTnI), lactate dehydrogenase (LDH), and aspartate transaminase (AST). All analyses were conducted according to the manufacturer's instructions (Jiancheng Bioengineering Institute, China).

### Enzyme-linked immunosorbent assay (ELISA)

The content of inflammatory factors interleukin (IL)-1β, IL-6, and tumor necrosis factor (TNF)-α in myocardial tissues were detected using commercial ELISA kits (Beyotime Biotechnology). All the experimental procedures were carried out according to the instructions.

### Histopathological analysis

The isolated myocardial tissues were immediately fixed in 4% paraformaldehyde, embedded in paraffin, and transversely cut into thin sections (5 μm). The myocardial sections were exposed to the hematoxylin-eosin (HE) staining procedure according to the manufacturer's instructions (Solarbio, China). Micrographs of ventricular myocardium tissue slides were taken under a light microscope (Olympus, Japan) at 200× magnification. The slides were evaluated in a blinded manner by a board-certified pathologist.

### RNA-sequencing

Total RNA was extracted from the isolated myocardial tissues using TRIzol reagent and was quantified by spectrophotometry. cDNA libraries were prepared according to the standard Illumina protocol, and the RNA-sequencing process and data analysis were completed on an Illumina NovaSeq 6000 platform provided by Metware Biotechnology Co., Ltd. (China). Data normalization was carried out via the variance stabilizing transformation function in DESeq2. The experiment was conducted with six biological replicates per experimental group. The criteria for screening significant genes were P<0.05 and |log_2_ FC| >1.

### Transmission electron microscope (TEM)

After incubation in exclusive electron microscope fixative overnight (12-16 h), the myocardial tissues were fragmented into small pieces and fixed in 2% osmium tetroxide and 2% glutaraldehyde for at least 2 h. Afterwards, the fixed myocardial tissues were embedded in Epon 812 resin (Beijing XXBR Technology Co., Ltd., China). The embedded myocardial tissues were then processed to prepare ultrathin sections according the standard protocols and the subcellular structure was observed under a transmission electron microscope at 80 kV (Hitachi, Japan).

### Immunohistochemical staining

The aforementioned myocardial sections underwent antigen retrieval procedures, were treated with the primary rabbit anti-Beclin 1 antibody (Abcam, UK) overnight at 4°C, and immersed in HRP-labeled goat anti-rabbit IgG (1:50, ThermoFisher, USA) at 37°C for 1 h. Diaminobenzidine slide (Solarbio) staining and hematoxylin (Solarbio) counterstaining was then carried out. The target protein was visualized under a light microscope (Olympus) at 200× magnification. Three fields of view were randomly selected for photographing each section.

### Cell culture and treatment

#### Cell culture

H9c2 cells (rat cardiomyoblasts) were purchased from Zhong Qiao Xin Zhou Biotechnology Co., Ltd. (China) and were cultured in DMEM medium (Solarbio) supplemented with 10% fetal bovine serum (Tianhang Biotechnology Co., Ltd., China) at 37°C with 5% CO_2_ and 95% air.

H9c2 cells were exposed to liquiritin at different concentrations (0.001, 0.01, 0.1, 1, 10, 20, 40, and 80 μM) for 48 h following cell viability detection using CCK8 assay.

#### OGD/R procedure

H9c2 cells were pre-treated with 10 or 20 μM liquiritin for 24 h. Then, cells were transferred to glucose-free DMEM medium and incubated in a hypoxic incubator (0.5% O_2_, 94.5% N_2_ and 5% CO_2_ at 37°C) for 6 h. Afterwards, complete DMEM medium was applied and the culture environment was adjusted as the standard (5% CO_2_ and 95% air at 37°C) for the next 18 h before the cells were harvested.

#### PIK3CA knockdown

H9c2 cells were transfected with PIK3CA siRNA using Lipofectamine 3000 according to the manufacturer's instructions (Generalbiol, China). Control siRNA (siNC) transfection served as control. After 24 h, transfection efficiency was determined by detecting PIK3CA protein expression. The transfected H9c2 cells were subjected to 20 μM liquiritin pretreatment and OGD/R procedure.

#### Chloroquine treatment

H9c2 cells were pre-treated with liquiritin (10/20 μM) for 24 h following the aforementioned OGD/R procedure. Chloroquine (20 μM, MedChem Express, USA) was administered 4 h before OGD/R treatment.

### CCK8 assay

After the OGD/R procedure, liquiritin-treated H9c2 cells were subjected to CCK8 assay to measure cell viability. The cells were incubated in 10 μL CCK-8 working solution (Solarbio) at 37°C for 2 h following absorbance determination at 450 nm by a microplate reader (BIOTEK, USA).

### Immunofluorescence

The aforementioned myocardial sections underwent dewaxing, rehydration, and antigen retrieval procedures, and H9c2 cells were permeabilized with TritonX-100. The samples were incubated in rabbit anti-PIK3CA or LC3B primary antibody (dilution: 1:100, Abcam, USA) at 4°C overnight and in Alexa Flour594 goat anti-rabbit IgG (ThermoFisher Scientific) at room temperature for 1 h. The nuclei were counterstained by DAPI (Aladdin, China). Finally, the positive cells were observed under a fluorescence microscope at a magnification of 400×.

### TUNEL staining

Apoptosis in myocardial tissues and treated H9c2 cells was detected by TUNEL assay using an *in situ* cell death detection kit (Roche, Switzerland). All the experimental procedures were in accordance with the manufacture's instruction of Terminal deoxynucleotidyl transferase-mediated dUTP (2-deoxyuridine 5-triphosphate) nick-end labeling (TUNEL) assay. The nuclei of H9c2 cells were counterstained by DAPI (Aladdin). The apoptosis-positive cells in the myocardial tissues were observed under a light microscope (Olympus) at 200× magnification, while the apoptotic H9c2 cells were counted under a fluorescence microscope (Olympus) at 400× magnification. The TUNEL images were analyzed to quantify the percentage of apoptotic cells using software ImageJ (NIH, USA).

### Western blot analysis

The proteins from myocardial tissues or cells were extracted using cell RIPA lysis buffer (Solarbio) with protease inhibitor PMSF (Solarbio), and the protein concentration was determined by a BCA kit (Solarbio). After being loaded on the sodium dodecyl sulfate polyacrylamide gel (SDS-PAGE), the protein was separated based on the molecular weight by electrophoresis following transfer to polyvinylidene difluoride (PVDF) membranes. The PVDF membrane was blocked with 5% skimmed milk for 2 h at room temperature. The membrane was incubated in primary antibody overnight (12-16 h) and homologous HRP-labelled secondary antibody (dilution: 1:2000, Abclonal, China) for 2 h. The protein bands were visualized using a chemiluminescence (ECL) kit (Beyotime Biotechnology). Finally, the integrated intensity of visualized bands was calculated by Gel-Pro-Analyzer (Media Cybernetics, Inc., USA).

The primary antibodies were as follows: rabbit anti-Bax, anti-Bcl-2, anti-cleaved caspase-3, anti-cleaved caspase-9, anti-beclin1, anti-p-PI3K, anti-PI3K, anti-p-AKT, anti-AKT, anti-p-mTOR, anti-mTOR (dilution: 1: 1000, Abclonal), anti-PIK3CA (dilution: 1:1000, Abcam), and GAPDH (dilution: 1:50000, Abclonal).

### Statistical analysis

Data are reported as means±SD. The data were tested for normality using the Shapiro-Wilk test and found to conform to a normal distribution (P>0.05). The data were analyzed by one-way ANOVA followed by Tukey's multiple comparison test by GraphPad Prism 8.0 software (USA). P<0.05 was considered statistically significant. The *in vitro* assays were repeated three times (n=3) and the *in vivo* experiments were repeated six times (n=6). All samples were subjected to triplicate testing to ensure the accuracy and reliability of the results.

## Results

### Liquiritin provided cardioprotective effects in myocardial I/R insulted rats

**Figure 1:**
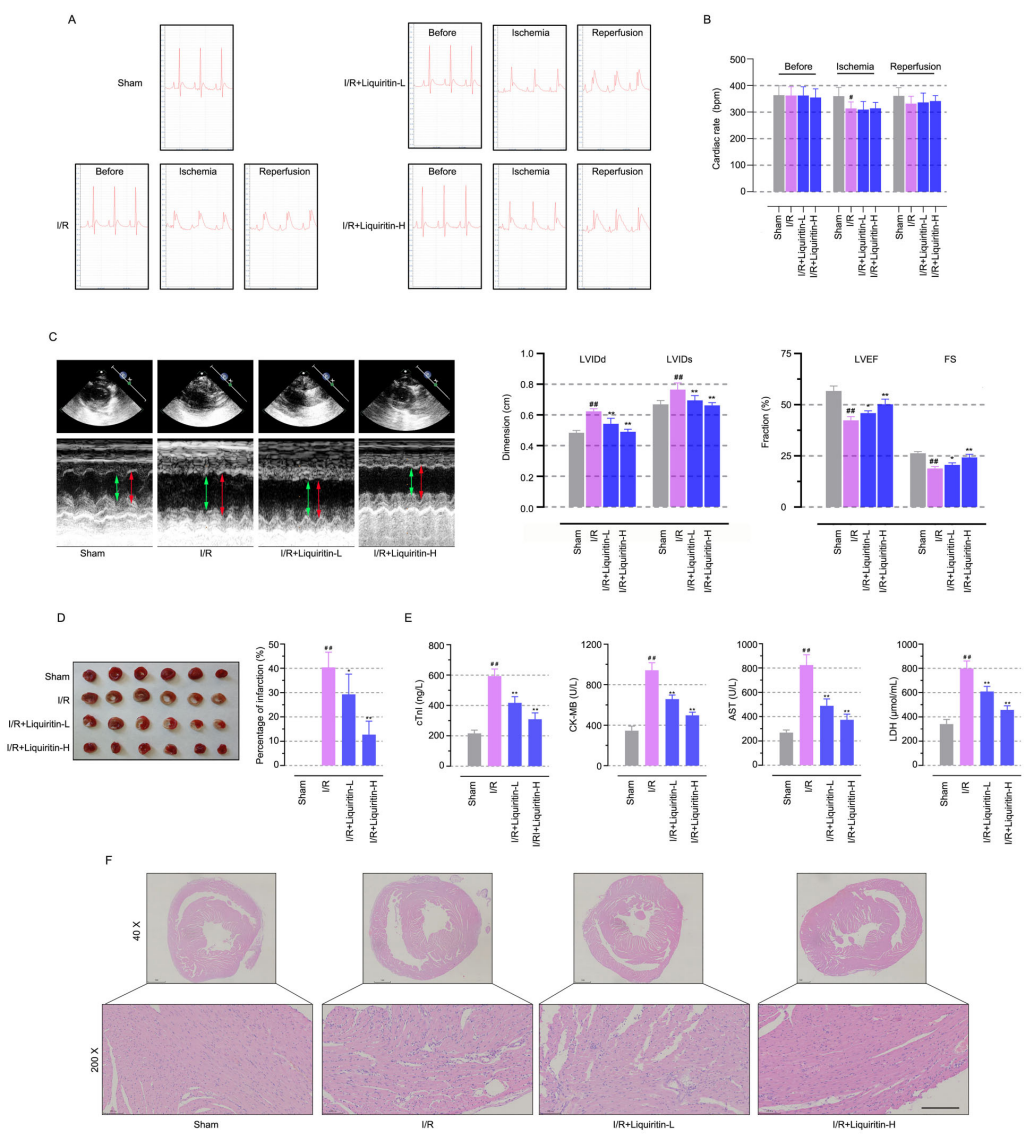
Liquiritin provided cardioprotective effect in myocardial ischemia-reperfusion (I/R) insulted rats. **A**, Representative images of echocardiogram (ECG) graphs. **B**, Change in heart rate. **C**, The ECG images, dimension, and fraction. **D**, Representative images of TTC staining and infarct size quantification. **E**, Levels of myocardial cTnI, CK-MB, AST, and LDH after liquiritin treatment. **F**, Representative images of H&E staining of myocardial tissues (scale bar=100 μm). Data are reported as means±SD (n=6) and were analyzed by one-way ANOVA followed by Tukey's multiple comparison test. ^##^P<0.01 *vs* the Sham group and *P<0.05, **P<0.01 *vs* the I/R group.

As shown in Figure 1A, typical ECG changes, including obvious ST-segment elevation and arrhythmias were present in I/R injured rats, and liquiritin treatment contributed to the restoration of normal ECG after I/R injury. Liquiritin mitigated heart rate reduction resulting from I/R injury (Figure 1B). Figure 1C shows that I/R injury decreased LVEF (42.42±1.71 *vs* Sham: 56.80±2.37, P<0.0001) and FS (18.85±0.90 *vs* Sham: 26.37±0.73, P<0.0001), while increasing LVIDd (0.62±0.02 *vs* Sham: 0.48±0.01, P<0.0001) and LVIDs (0.76±0.04 *vs* Sham: 0.66±0.3, P=0.0002). Liquiritin treatment (20/40 mg/kg) significantly improved LVEF (45.92±1.11/50.23±2.5 *vs* I/R, P=0.0324/P<0.0001) and FS (20.62±1.04/24.30±1.46 *vs* I/R, P=0.0443/P<0.0001), with reduced LVIDd (0.54±0.03/0.49±0.02 *vs* I/R, P=0.0443/P<0.0001) and LVIDs (0.69±0.03/0.66±0.02 *vs* I/R, P=0.0047/P<0.0001). The images of TTC staining were used to evaluate the myocardial infarct scope. As shown in Figure 1D, obvious infarction was found in myocardial tissues after I/R insult compared with the sham rats (40.36±6.27 *vs* Sham: 0, P=0.0001), while the administration of liquiritin (20/40 mg/kg) significantly reduced the infarct size (29.32±8.31/12.76±5.50 *vs* I/R, P=0.0375/P=0.0019). The levels of myocardial injury markers, including cTnI, CK-MB, AST, and LDH, showed evidence of the effect of liquiritin on myocardial I/R injury. The biochemical detection demonstrated that liquiritin down-regulated these abnormally elevated indexes (Figure 1E, P<0.01). Consistent with elevated cardiac function after liquiritin treatment, the histopathological examination results suggested that pathological changes in cardiac tissues were significantly reversed after treatment with liquiritin. (Figure 1F). These results indicated that liquiritin possessed cardioprotective functions in the myocardial I/R-injured models.

### Liquiritin suppressed apoptosis in the myocardium with I/R injury

**Figure 2:**
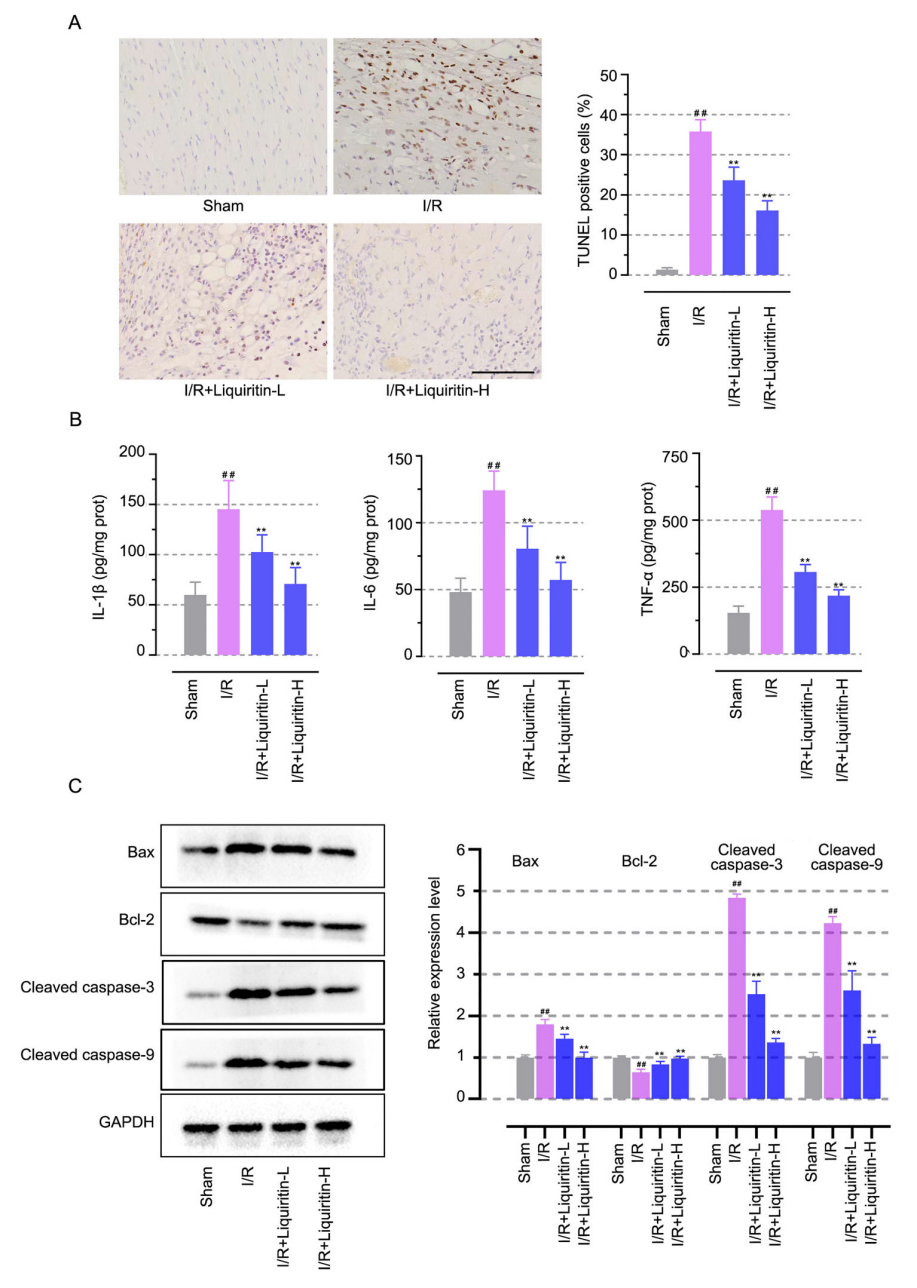
Liquiritin suppressed apoptosis in the myocardium with ischemia-reperfusion (I/R) injury. **A**, Representative images of myocardial TUNEL staining and apoptosis quantification (scale bar=100 μm). **B**, Levels of myocardial interleukin (IL)-1β, IL-6, and tumor necrosis factor (TNF)-α after liquiritin treatment. **C**, Western blot analysis of Bax, Bcl-2, cleaved caspase-3, and cleaved caspase-9 levels. Data are reported as means±SD (n=6) and were analyzed by one-way ANOVA followed by Tukey's multiple comparison test. ^##^P<0.01 *vs* the Sham group and **P<0.01 *vs* the I/R group.

To elucidate the effect of liquiritin on the myocardium with I/R injury in detail, we assessed inflammation and apoptosis simultaneously in the myocardium exposed to I/R injury. The release of pro-inflammatory factors related to apoptosis induction, such as IL-1β, IL-6, and TNF-α, were evaluated in the myocardial I/R injured models. As indicated in Figure 2A, compared with sham surgery treatment, myocardium with I/R injury induced an upregulation of TUNEL-positive cells, which was diminished by liquiritin administration (P<0.01). Liquiritin treatment significantly reduced the secretion of pro-inflammatory cytokines (Figure 2B, P<0.01). Consistently, the expression of apoptosis-related proteins showed that treatment with liquiritin down-regulated pro-apoptotic protein Bax, cleaved caspase-3, and cleaved caspase-9 levels, while it up-regulated anti-apoptotic Bcl-2 in the cardiac tissues (Figure 2C, P<0.01). These results demonstrated that liquiritin inhibited apoptosis induced by I/R in the myocardium.

### Liquiritin regulated PIK3CA expression in the myocardium with I/R injury

**Figure 3:**
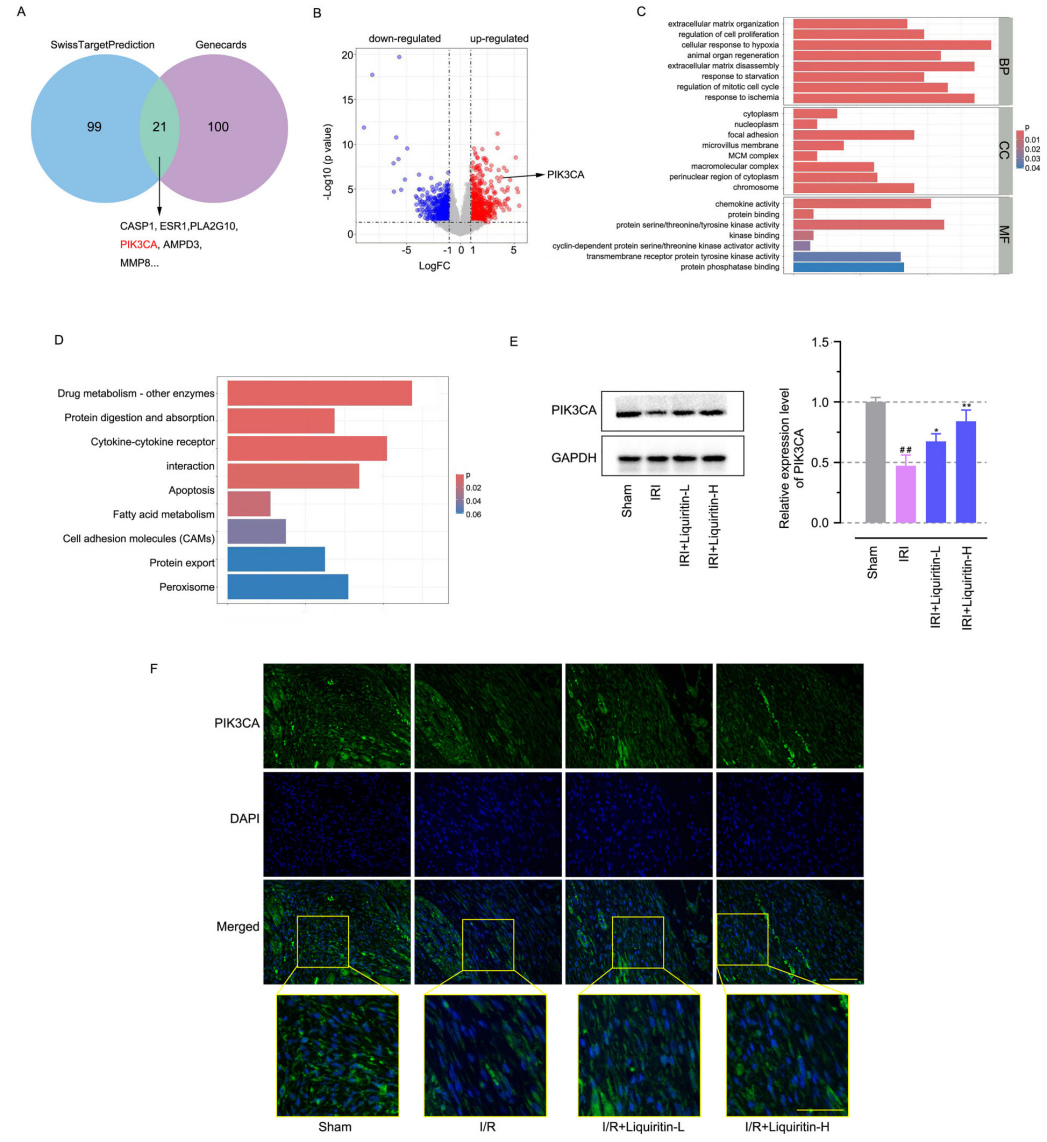
Liquiritin regulated PIK3CA expression in the myocardium with ischemia-reperfusion (I/R) injury. **A**, Potential targets of liquiritin for its cardioprotective effects against myocardial I/R injury. **B**, Volcano plot showing the dysregulated mRNAs. **C**) The GO functional analysis and (**D**) KEGG pathway analysis. **E**, Western blot analysis of PIK3CA levels in the myocardial tissues. **F**, Immunofluorescence targeting PIK3CA in the myocardial tissues of rats with liquiritin administration (scale bar=50 μm). Data are reported as means±SD (n=6) and were analyzed by one-way ANOVA followed by Tukey's multiple comparison test. ^##^P<0.01 *vs* the Sham group and *P<0.05, **P<0.01 *vs* the I/R group.

We then investigated potential targets of liquiritin for its cardioprotective effects against myocardial I/R injury. The platform SwissTargetPrediction (http://www.swisstargetprediction.ch/) estimated the most probable macromolecular targets of liquiritin and the Genecards database (https://www.genecards.org/) provided the predicted genes related to myocardial I/R injury. We assessed the potential target of liquiritin on myocardial I/R injury by intersecting these two predicted results and there were 21 potential predicted targets in total, including PIK3CA (Figure 3A). The next RNA-sequencing results indicated that PIK3CA was significantly dysregulated and modulated by liquiritin treatment compared with the I/R injured myocardium (Figure 3B). Gene Ontology (GO) functional analysis and Kyoto Encyclopedia of Genes and Genomes (KEGG) pathway analysis further demonstrated that PIK3CA might be the potential target mediating the cardioprotective effects of liquiritin (Figure 3C and D). Western blot and immunofluorescence targeting PIK3CA were carried out. Consistent with the transcriptomics results, the protein expression of PIK3CA in the myocardium was markedly reduced when exposed to I/R injury and the reduction was restored by liquiritin (Figure 3E and F, P<0.05), the mechanism of which was explored in the following experiments.

### Liquiritin was involved in restoring abnormal autophagy related to myocardial I/R injury

**Figure 4:**
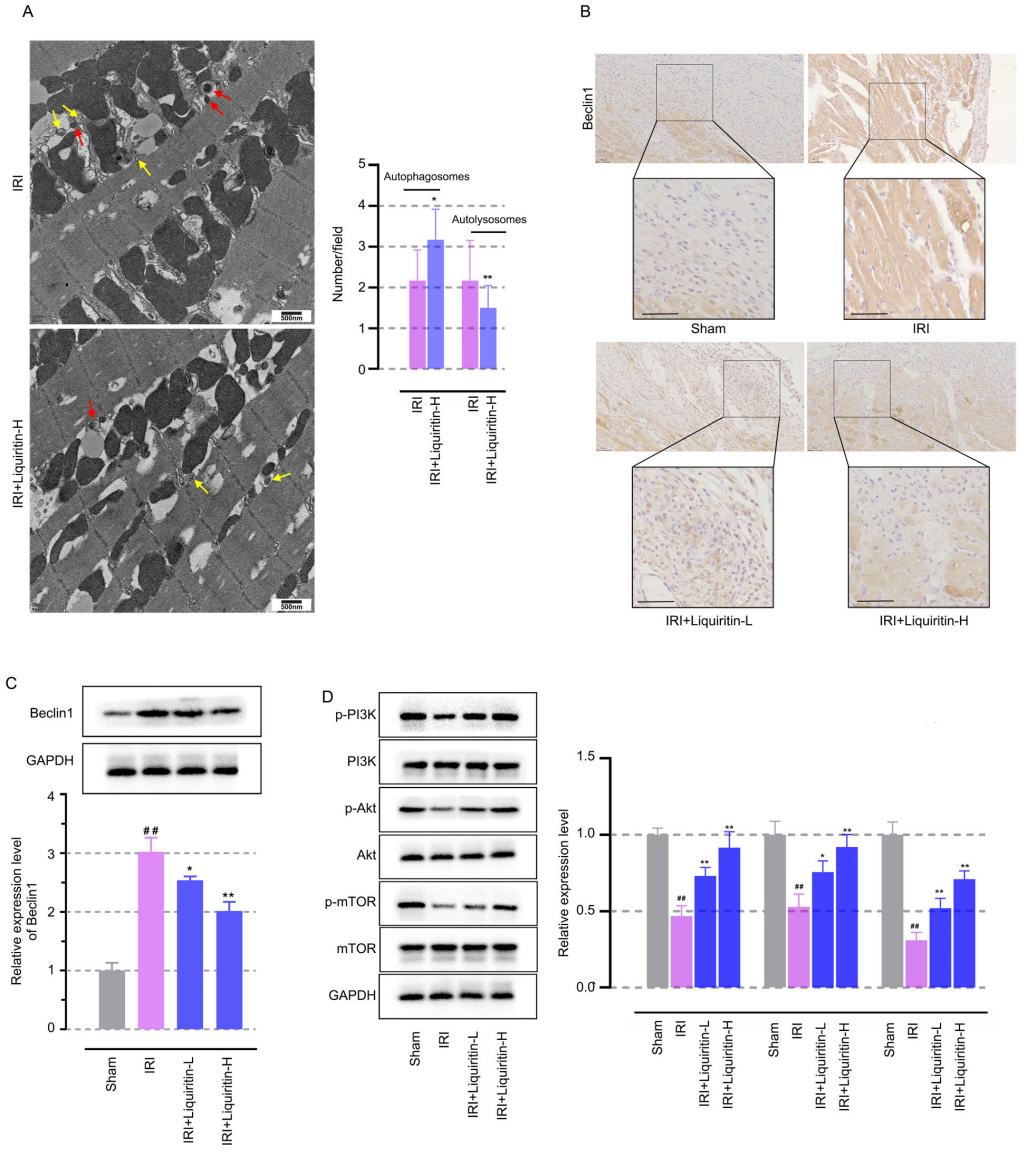
Liquiritin involved in restoring abnormal autophagy related to myocardial ischemia-reperfusion (I/R) injury. **A**, Representative images of TEM in I/R injured myocardial tissues of rats after liquiritin administration. Yellow arrow: autophagosomes, red arrow: autolysosomes (scale bar=500 nm). **B**, Immunohistochemistry targeting autophagy marker Beclin1 in the myocardial tissues (scale bar=100 μm). **C**, Western blot analysis of Beclin1 levels in the myocardial tissues. **D**, Western blot analysis of p-PI3K, p-Akt, and p-mTOR levels in the myocardial tissues. Data are reported as means±SD (n=6) and were analyzed by ANOVA followed by Tukey's multiple comparison test. ^##^P<0.01 *vs* the Sham group and *P<0.05, **P<0.01 *vs* the I/R group.

Considering the abovementioned pathway analysis results, we next examined the changes in autophagy after liquiritin administration *in vivo*. The TEM results show that compared to the I/R-treated myocardial tissues, liquiritin administration led to an increase in autophagosomes and a decrease in autolysosomes, while alleviating mitochondrial damage, suggesting that inhibiting excessive autophagic flux by reducing autophagosome-lysosome fusion might contribute to its protective effects (Figure 4A, P<0.05). As shown in Figure 4B and C, results from immunohistochemical and western blot detection targeting autophagy marker Beclin1 suggested that autophagy was aggravated after myocardial I/R injury (P<0.05), and this phenomenon was reversed by liquiritin, accompanied by the recovery of abnormal activation of the PI3K/Akt/mTOR pathway (Figure 4D, P<0.05). These results showed that liquiritin inhibited excessive autophagy by restoring the PI3K/Akt/mTOR pathway.

### Liquiritin protected *H9c2* cells from OGD/R injury by increasing PIK3CA expression

**Figure 5:**
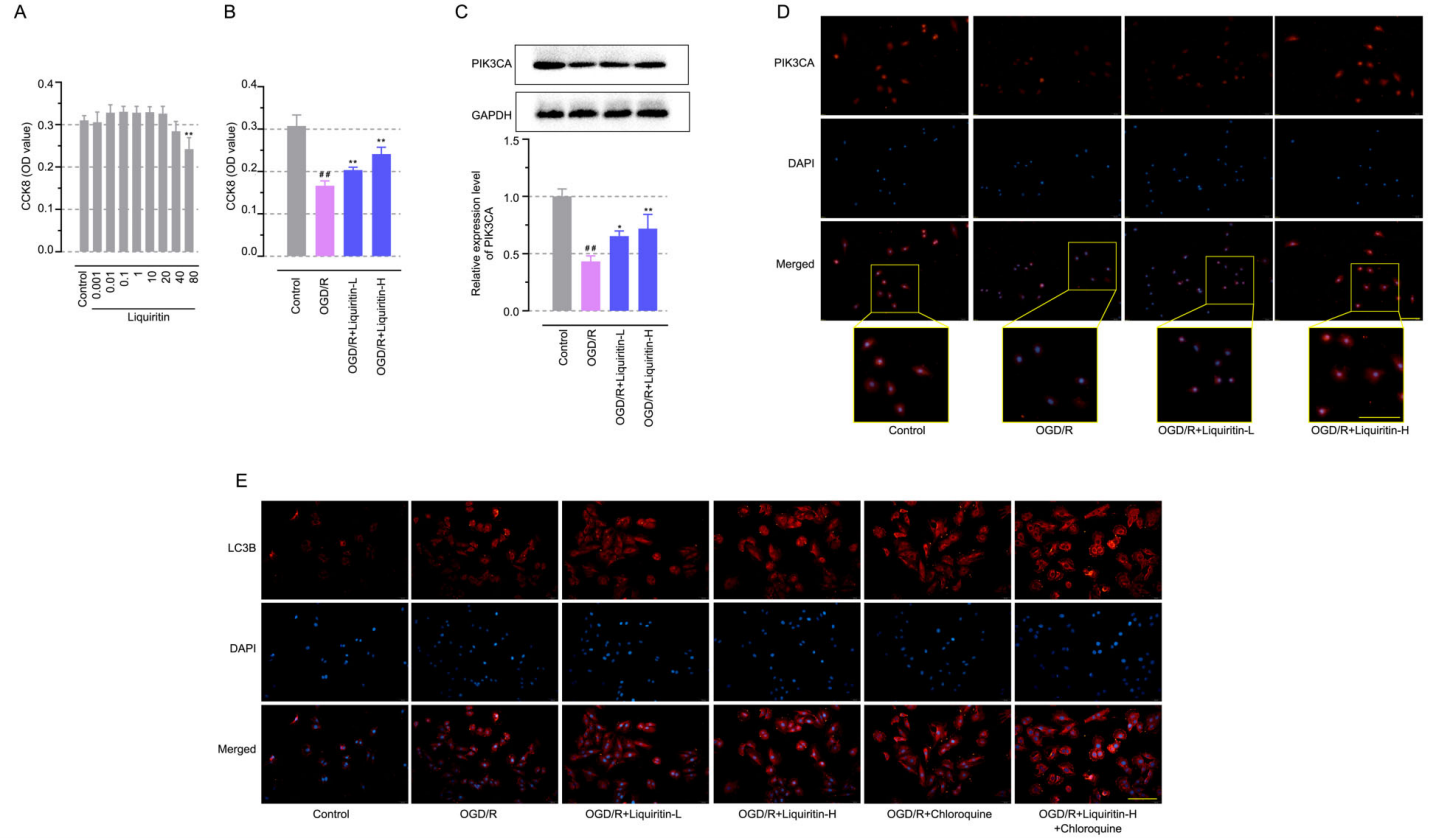
Liquiritin protected *H9c2* cells from oxygen-glucose deprivation/reoxygenation (OGD/R) injury via increasing PIK3CA expression. **A**, CCK8 assay was used to assess proliferation ability in *H9c2* cells exposed to liquiritin at different concentrations (0.001-80 μM). **B**, CCK8 assay was used to assess proliferation ability in OGD/R injured *H9c2* cells. **C**, Western blot analysis of PIK3CA levels. **D**, Immunofluorescence targeting PIK3CA (scale bar=50 μm). **E**, Immunofluorescence targeting LC3B in OGD/R injured *H9c2* cells after liquiritin and/or chloroquine (20 μM) treatment. (scale bar=50 μm). Data are reported as means±SD (n=3) and were analyzed by ANOVA followed by Tukey's multiple comparison test. ^##^P<0.01 *vs* the Control group and *P<0.05, **P<0.01 *vs* the OGD/R group.

The expression of PIK3CA was detected in OGD/R-injured H9c2 cells. Initially, the CCK-8 assay was used to screen the *in vitro* doses of liquiritin (Figure 5A, P<0.05). Liquiritin at 0-40 µM exhibited no significant cytotoxicity, but a downward trend was observed at 40 µM. Hence, 10 and 20 liquiritin µM were selected for the following experiment. As shown in Figure 5B, liquiritin reversed OGD/R-induced cell viability decrease (P<0.01) and liquiritin reversed PIK3CA reduction induced by OGD/R in H9c2 cells (Figure 5C and D, P<0.05). Afterwards, chloroquine inhibited autophagic flux via decreasing autophagosome-lysosome fusion, which was used to evaluate the autophagic machinery in which liquiritin was involved. As expected, the increased fluorescence intensity of LC3B after liquiritin administration could be attributed to enhanced autophagosome formation or autophagic flux inhibition. Interestingly, this increase was similar in magnitude to that caused by chloroquine, an inhibitor of autophagosome-lysosome fusion (Figure 5E). Notably, co-treatment with liquiritin and chloroquine further enhanced LC3B fluorescence, indicating an additive effect on autophagic flux inhibition (Figure 5E). The data suggested that liquiritin might promote autophagy restoration by inhibiting autophagosome-lysosome fusion.

### Liquiritin restored autophagy via the PI3K/Akt/mTOR pathway mediated by PIK3CA in OGD/R-treated H9c2 cells

**Figure 6:**
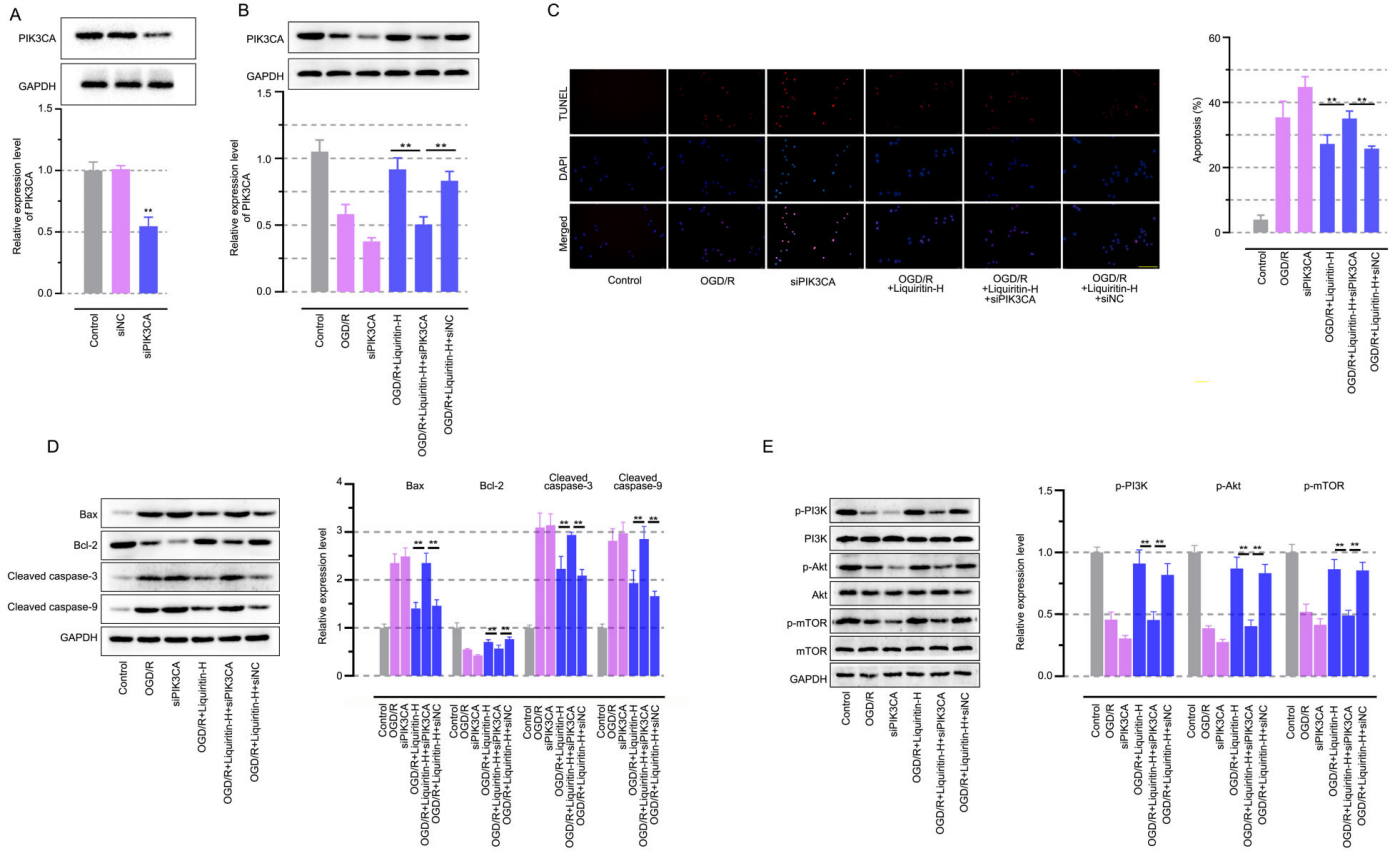
Liquiritin restored autophagy via the PI3K/Akt/mTOR pathway mediated by PIK3CA in oxygen-glucose deprivation/reoxygenation (OGD/R)-treated *H9c2* cells. **A**, Western blot analysis of PIK3CA levels in *H9c2* cells with transfection. **B**, Western blot analysis of PIK3CA levels. **C**, Representative images of TUNEL staining and apoptosis quantification (scale bar=50 μm). **D**, Western blot analysis of Bax, Bcl-2, cleaved caspase-3, and cleaved caspase-9 levels. **E**, Western blot analysis of p-PI3K, p-Akt, and p-mTOR levels. Data are reported as means±SD (n=3) and were analyzed by one-way ANOVA followed by Tukey's multiple comparison test. ^##^P<0.01 *vs* the Control group and **P<0.01 *vs* the OGD/R group.

To investigate whether the protective effect of liquiritin on myocardial I/R injury restoration was mediated via regulating PIK3CA-related autophagy, we partially silenced PIK3CA in H9c2 cells (Figure 6A and B, P<0.01). As shown in Figure 6C, liquiritin protected H9c2 cells from OGD/R induced apoptosis, evidenced by TUNEL assay, while the phenomenon could be abolished by PIK3CA knockdown. The knockdown of PIK3CA was used to show higher damage compared to that observed in the OGD/R model. Similarly, western blot assay showed increased bax, cleaved caspase-3, and cleaved caspase-9 and decreased bcl-2 expression in H9c2 cells after PIK3CA siRNA transfection (Figure 6D, P<0.01). Silencing PIK3CA abolished liquiritin's restoration of the abnormal PI3K/Akt/mTOR signaling pathway, which is crucial for the regulation of autophagy (Figure 6E, P<0.01). Collectively, these *in vitro* findings coupled with the above-mentioned *in vivo* results demonstrated that the autophagy regulation of the PI3K/Akt/mTOR pathway mediated by PIK3CA was essential for liquiritin's protection against myocardial I/R injury.

## Discussion

At present, the conventional treatment approaches for patients with myocardial infarction include percutaneous coronary intervention and surgical coronary artery bypass grafting, both of which help restore coronary perfusion. Although these options limit the ischemic zone of the myocardium, the subsequent damage caused by reperfusion cannot be ignored. Notably, despite decades of research, no single drug has been established as a standard, universally-approved treatment for myocardial I/R injury in clinical practice. Therefore, searching for therapeutic drugs and suitable targets for controlling myocardial I/R injury plays a critical role in reducing the morbidity rate of myocardial infarction. In the present study, the administration of liquiritin provided cardioprotective effects in myocardial I/R insulted rats, which was evidenced by reduced infarct size and improved cardiac function. Furthermore, liquiritin reduced inflammation in myocardial tissues and displayed an anti-apoptosis effect after I/R injury *in vivo*. Our current results showed that liquiritin was involved in restoring abnormal autophagy related to myocardial I/R injury, reflected by autophagic markers. Furthermore, the decreased expression of PIK3CA in myocardial tissues caused by I/R injury was restored by liquiritin treatment. Mechanistically, these beneficial effects of liquiritin were abrogated by PIK3CA knockdown in OGD/R injured *H9c2* cells, indicating that the regulatory role of liquiritin on autophagy restoration was mediated by PIK3CA expression. Hence, we speculated that liquiritin was involved in cardioprotective effects by regulating abnormal autophagy mediated by PIK3CA in myocardial I/R injury models, which might offer a novel perspective in improving the treatment strategy targeting myocardial infarction.

To our knowledge, licorice root is a commonly employed herbal medicine in China, with flavonoids being the major bioactive components, such as liquiritin and isoliquiritin (23). Previous studies have reported the potential of liquiritin on cardiomyocyte injury or fibrosis (29), but its role in myocardial I/R injury still requires further investigation. Recent research has indicated potential targets and mechanisms of liquiritin in the treatment of myocardial I/R injury based on network pharmacology. *In vitro* experiments in an OGD/R injured *H9c2* cell model indicated that the positive role is achieved via regulating TNF-α receptor type 1 (TNFR1)/NF-κB/MMP9 pathway (28). Nevertheless, the efficacy of liquiritin in treating myocardial I/R injury at the *in vivo* level still requires further investigation, and the underlying mechanism also remains unclear.

In the present study, we firstly validated whether liquiritin possessed a protective effect against myocardial I/R injury in rats. It is well accepted that TTC staining is a rapid, efficient, and widely used method for assessing the extent of infarction. TTC, a colorless and lipid-soluble chemical agent, can be restored by mitochondrial enzymes into a compound, which dyes the normal myocardial tissue red, while the infarcted tissue remains white. By assessing the myocardial infarct volume using TTC staining, we found that the treatment of liquiritin significantly decreased infarct volume following myocardial I/R injury, indicating its potential benefits. However, no previous study has indicated the potential mechanism involved.

Current theories have postulated that autophagy is a key regulator of cardiac homeostasis and function (30). The term autophagy was coined by Nobel laureate Christian de Duve in 1963, who hypothesized the existence of cellular machinery to transport cytosolic constituents to lysosomes for digestion. Autophagy plays a crucial role in maintaining cardiac structure and function under normal conditions and is triggered during stress to minimize damage (31,32). Autophagy activation involves injury reduction and cardiac function preservation during ischemia. It also inhibits myocardial remodeling induced by chronic ischemia and elevates the cardiac adaptation ability to pressure overload via disintegrating misfolded proteins, restoring mitochondrial function, and preventing oxygenated stress damage (33). Although autophagy deficiencies play a role in the emergence of cardiac proteinopathy and doxorubicin-induced cardiomyopathy, the excessive activation of autophagy has negative effects on the heart under specific stress circumstances, such as I/R injury (16). As previously reported by Qin et al. (13), ginsenoside Rb1 served as a protective agent against myocardial I/R injury by restraining excessive autophagy in OGD/R-injured cardiomyocytes via the PI3K/Akt/mTOR signaling pathway. As expected, the administration of liquiritin inhibited excessive autophagy *in vivo*. To actually demonstrate the possible mechanism by which liquiritin restored abnormal autophagy, we used chloroquine to block the autophagic flux at late stage. Consistent with our expectations, liquiritin inhibited excessive autophagy by suppressing autophagosome-lysosome fusion in OGD/R-treated *H9c2* cells.

Irreversible apoptosis is the cause of cardiomyocyte damage resulting from myocardial ischemia and is recognized as the primary contributor to myocardial cell loss, and excessive autophagy has been demonstrated to contribute to this phenomenon (30). A previously published work (17) briefly stated that cardiomyocyte autophagy activation could be rapidly triggered in the early stages of myocardial I/R injury and be involved in limiting the insult. However, that study also indicated that autophagy had a double function when I/R injury occurs. During the early stage of myocardial I/R injury, controlled or adaptive autophagy eliminates damaged components to maintain cell viability. However, its further enhancement in the reperfusion phase may degrade essential proteins and organelles, which triggers autophagic cardiomyocyte death, exacerbating myocardial injury (17). Studies by He et al. (34) demonstrated that dexmedetomidine inhibited oxidative stress and inflammatory response in the I/R-injured myocardial tissues and elevated autophagic flux, thereby protecting hearts from I/R injury. On the contrary, resveratrol was reported to alleviate myocardial I/R injury by suppressing excessive autophagy via the DJ-1/MEKK1/JNK pathway (31). Similarly, tanshinone IIA contributed to myocardial injury protection, which was evidenced by improving energy supply, balancing excessive autophagy, as well as alleviating apoptosis (32). As mentioned above, we also found that the administration of liquiritin protected I/R-insulted cardiomyocytes from dysregulated autophagy and aberrant apoptosis, but the precise mechanism of liquiritin on myocardial I/R injury had not been elucidated.

**Figure 7:**
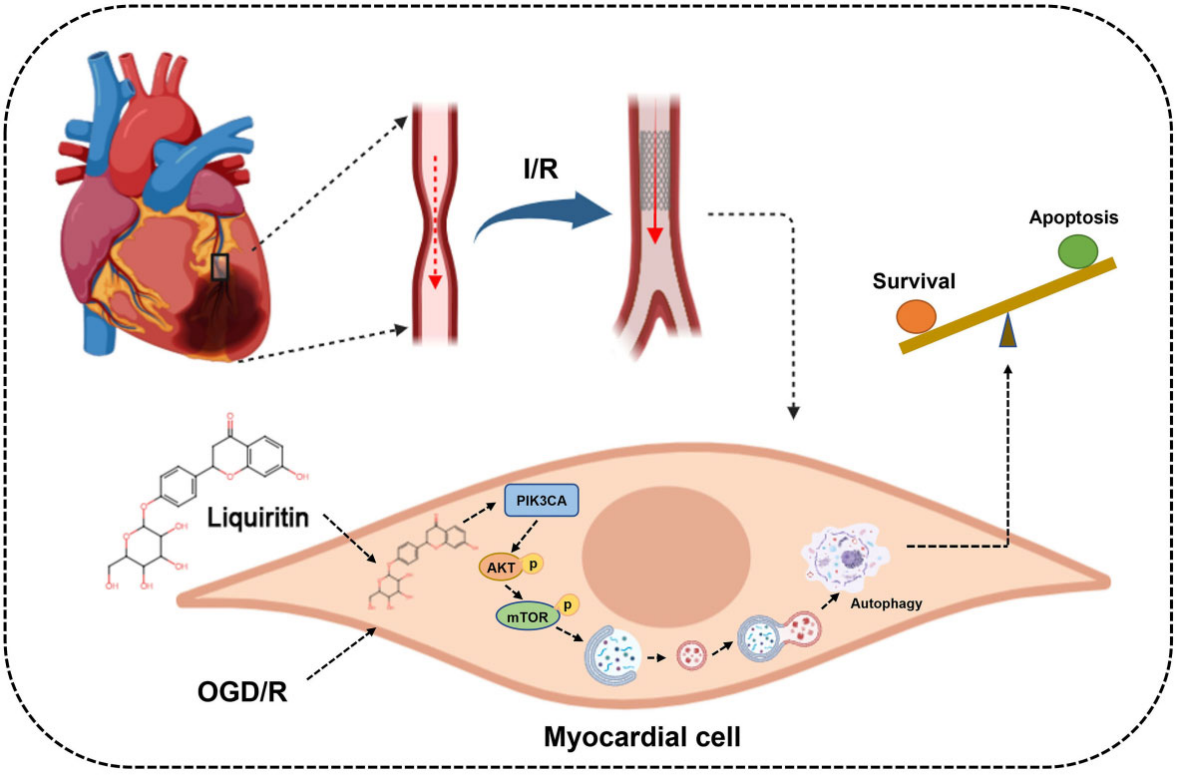
Mechanism of action of liquiritin treatment in PIK3CA expression, consequently restoring dysfunctional autophagy through the PI3K/Akt/mTOR pathway. I/R: ischemia-reperfusion; OGD/R: oxygen-glucose deprivation/reoxygenation.

A recent study has found that the PIK3CA signaling pathway is a vital regulator of myocardial structure and function in both developmental and pathophysiological conditions (35). Gong et al. (36) has reported that local administration of the small-molecule PIK3CA activator, UCL-TRO-1938, confers cardioprotection against I/R injury. Meanwhile, it is well understood that accelerated cancer progression can be attributed to excessive activation of PI3K subunit, including PIK3CA, and many PI3K inhibitors are used for cancer therapies in clinical trials (37). The systemic effects of PI3K inhibition, including cardiotoxicity, may contribute to the occurrence of cardiac damage in anti-tumor therapies (36). In addition, blocking PI3K attenuates the protective effects of growth factors and other agents in models of cell and tissue damage, including myocardial I/R injury (36). Our findings showed that PIK3CA expression was low in myocardial tissue after I/R insult, which could be reversed by liquiritin treatment. Consistent observations were made in the OGD/R injured *H9c2* cell model: the therapeutic effect of liquiritin was weakened or even abolished when PIK3CA was silenced, indicating that liquiritin alleviated cardiac damage by promoting PIK3CA expression. PIK3CA is essential for PI3K activity, and the PI3K/Akt/mTOR pathway is an important cellular signaling that is essential for fundamental intracellular functions (38). Considering the aforementioned study, we preliminarily verified that liquiritin administration was involved in the regulation of the PI3K/Akt/mTOR pathway, suggesting that the classical PI3K/Akt/mTOR pathway was related to PIK3CA downstream. These data have allowed us to comprehend that liquiritin treatment regulates PIK3CA expression, consequently restoring dysfunctional autophagy through the PI3K/Akt/mTOR pathway, which is aligning with our initial expectations (Figure 7).

Collectively, liquiritin demonstrated significant cardioprotective effects in myocardial I/R insulted rats and OGD/R injured *H9c2* cells, indicating the potential to be developed as an adjunct therapy for patients at risk of myocardial ischemia in the future. In addition, liquiritin showed a positive impact on inhibiting excessive autophagy through the PI3K/Akt/mTOR pathway by activating PIK3CA.

## Data Availability

All data generated or analyzed during this study are included in this published article.
